# Analysis of clinical evidence on traditional Chinese medicine for the treatment of diabetic nephropathy: a comprehensive review with evidence mapping

**DOI:** 10.3389/fendo.2024.1324782

**Published:** 2024-03-27

**Authors:** Yating Gao, Zhenghong Li, Yiming Wang, Haoling Zhang, Ke Huang, Yujie Fu, Shanqiong Xu, Qingna Li, Xingfang Liu, Guangde Zhang

**Affiliations:** ^1^ Institute of Endocrinology, Xiyuan Hospital, China Academy of Chinese Medical Sciences, Beijing, China; ^2^ Graduate School, Beijing University of Chinese Medicine, Beijing, China; ^3^ Research Department, Swiss University of Traditional Chinese Medicine, Bad Zurzach, Switzerland; ^4^ Graduate School, China Academy of Chinese Medical Sciences, Beijing, China; ^5^ Postdoctoral Research Station, China Academy of Chinese Medical Sciences, Beijing, China; ^6^ Institute of Clinical Pharmacology, Xiyuan Hospital, China Academy of Chinese Medical Sciences, Beijing, China

**Keywords:** diabetes mellitus, diabetic complications, diabetic nephropathy, traditional Chinese medicine, randomized controlled trials, systematic review, evidence-based analysis, evidence mapping

## Abstract

**Objective:**

This study aims to map evidence from Randomized Controlled Trials (RCTs) and systematic reviews/Meta-analyses concerning the treatment of Diabetic Nephropathy (DN) with Traditional Chinese Medicine (TCM), understand the distribution of evidence in this field, and summarize the efficacy and existing problems of TCM in treating DN. The intention is to provide evidence-based data for TCM in preventing and treating DN and to offer a reference for defining future research directions.

**Methods:**

Comprehensive searches of major databases were performed, spanning from January 2016 to May 2023, to include clinical RCTs and systematic reviews/Meta-analyses of TCM in treating DN. The analysis encompasses the publishing trend of clinical studies, the staging of research subjects, TCM syndrome differentiation, study scale, intervention plans, and outcome indicators. Methodological quality of systematic reviews was evaluated using the AMSTAR (Assessment of Multiple Systematic Reviews) checklist, and evidence distribution characteristics were analyzed using a combination of text and charts.

**Results:**

A total of 1926 RCTs and 110 systematic reviews/Meta-analyses were included. The majority of studies focused on stage III DN, with Qi-Yin deficiency being the predominant syndrome type, and sample sizes most commonly ranging from 60 to 100. The TCM intervention durations were primarily between 12-24 weeks. Therapeutic measures mainly consisted of Chinese herbal decoctions and patented Chinese medicines, with a substantial focus on clinical efficacy rate, TCM symptomatology, and renal function indicators, while attention to quality of life, dosage of Western medicine, and disease progression was inadequate. Systematic reviews mostly scored between 5 and 8 on the AMSTAR scale, and evidence from 94 studies indicated potential positive effects.

**Conclusion:**

DN represents a significant health challenge, particularly for the elderly, with TCM showing promise in symptom alleviation and renal protection. Yet, the field is marred by research inconsistencies and methodological shortcomings. Future investigations should prioritize the development of standardized outcome sets tailored to DN, carefully select evaluation indicators that reflect TCM’s unique intervention strategies, and aim to improve the robustness of clinical evidence. Emphasizing TCM’s foundational theories while incorporating advanced scientific technologies will be essential for innovating research methodologies and uncovering the mechanisms underlying TCM’s efficacy in DN management.

## 1 Introduction

Diabetes Mellitus (DM) is a chronic disease severely jeopardizing human health, with approximately one-third of patients with DM advancing to Diabetic Nephropathy (DN) (1). In recent years, with the aging population, the prevalence of obesity, significant changes in dietary structures and lifestyles, the incidence of diabetes has been increasing (2, 3), becoming a major chronic disease affecting the quality of life and physical health of the elderly population. A recent survey by the International Diabetes Federation revealed that approximately 537 million adults (10.5%) worldwide were affected by diabetes in 2021, with projections suggesting that the global number of people with diabetes will reach 783 million by 2045 (4). China leads globally in DM prevalence and counts of elderly patients (5, 6). The global patient count is projected to reach 578 million by 2030 (7), with the highest incidence rates found amongst individuals aged 65 to 79 (8), constituting 50% of adult DM patients (6). Diabetic nephropathy, as a severe microvascular complication of DM, has a high mortality and morbidity rate, with an all-cause mortality rate approximately 30 times higher than that of DM patients without nephropathy (9). The structural damage to the kidneys and functional impairments worsen with the progression of DM, eventually leading to irreversible lesions that severely harm the patient’s physical health, affecting their normal life and work. It becomes a major cause of chronic kidney disease, end-stage renal disease (ESRD), and cardiovascular events (10, 11).

With the swift increase of DM cases, aging populations, and improvements in life quality, the incidence of DN in the elderly continues to rise (12), accounting for 46% of elderly diseases (13) and becoming a significant global health issue, thereby exacerbating the burden on societal, economic, and medical systems (14).

The onset of DN is insidious, and once it progresses to the stage of significant proteinuria, its progression speed to ESRD is approximately 14 times that of other renal diseases (15), becoming the leading cause of ESRD among middle-aged and elderly people in China. As the elderly experience physiological decline and weakened defense mechanisms, elderly DN patients present heterogeneity in clinical characteristics, blood glucose control targets, and choices of hypoglycemic agents. Thus, implementing effective intervention measures to delay renal disease progression is vital, and optimizing treatment outcomes is crucial.

Modern medical treatment of DN is predominantly symptomatic and lacks specificity and efficacy (16). On the other hand, TCM, grounded in a holistic approach, employs Chinese patent medicine, herbal compound formulas, and external therapies, offering individualized, multi-targeted, multi-pathway treatments. It exerts anti-inflammatory, antioxidative, and anti-apoptotic effects (17), inhibiting glomerular hypertrophy and sclerosis, protecting podocytes, delaying interstitial fibrosis, reducing proteinuria, creatinine, and urea nitrogen (18–20), improving clinical symptoms, delaying renal disease progression, and even reversing early DN (21). It has proven effective in enhancing patient quality of life and reducing the incidence of endpoint events (22), and with its stable efficacy, minimal side effects, and affordability (23), it plays a pivotal role in DN prevention and treatment (24).

Traditional Chinese Medicine (TCM) treatment emphasizes a holistic approach, offering personalized therapies based on the patient’s condition, medical history, psychological state, and environmental factors, aiming to improve the patient’s physical and mental imbalances and promote recovery from diseases. The clinical efficacy evaluation in TCM is based on a holistic view of the health state adjustment effect, encompassing comprehensive assessments including Western medicine efficacy, TCM syndrome efficacy, quality of life, patient-reported outcomes (PRO), and clinician-reported outcomes (CRO). It utilizes the TCM way of thinking, applying evidence-based medicine, epidemiology, and other modern clinical research methodologies to standardize and quantify the information obtained from the four TCM diagnostic methods, thereby forming an integrated evaluation system. This system provides objective efficacy feedback for TCM clinical practice (25).

Various clinical efficacy trials on Chinese patent medicines and herbal compound formulas for DN treatment have been conducted in clinical and scientific research work, and numerous systematic reviews and Meta-analyses have been published. However, the overall clinical evidence for DN treatment with different TCM classification measures is still unclear.

As a novel method of evidence integration, evidence mapping systematically combs through and comprehensively analyzes research in the concerned field, presenting evidence, advancements, and shortcomings of this research domain in a thorough, multi-dimensional manner (26, 27). It enhances the effectiveness and practicality of related research and optimizes scientific research resource allocation (28, 29). This study adopts an evidence map in a textual and graphical format to visualize the evidence distribution in the field of TCM treating DN (30), clarifying existing issues in current research, further refining the evidence system, and aiming to pinpoint directions for subsequent research.

## 2 Materials and methods

### 2.1 Search strategy

A systematic search was conducted across various databases: China National Knowledge Infrastructure (CNKI), WanFang, VIP Database, SinoMed, The Cochrane Library, EMbase, Web of Science, and PubMed to procure both Chinese and English literature regarding TCM in treating DN.

DN has always been a widely focused area with numerous related studies. Since 2016, there has been an increased attention towards the treatment of DN, particularly in the context of TCM. Therefore, we chose to start our search from that year. This strategy allowed us to select a significant number of recent and relevant publications, specifically focusing on the research of TCM treatments for DN from January 2016 to May 2023. This approach provides a comprehensive reference for future studies in this field.

Literature retrieval primarily combined subject words and free words, utilizing the following search strategy: TS=((“Diabetic Nephropathies”[MeSH Terms] OR “diabetic nephropathy”[Title/Abstract] OR “diabetic kidney disease”[Title/Abstract] OR “diabetic kidney diseases”[Title/Abstract] OR “diabetic glomerulosclerosis”[Title/Abstract]) AND (“medicine, Chinese traditional”[MeSH Terms] OR “traditional Chinese medicine”[Title/Abstract] OR “Chinese traditional medicine”[Title/Abstract] OR “zhong yi xue”[Title/Abstract] OR “acupuncture”[Title/Abstract] OR “herbal medicine”[Title/Abstract] OR “Chinese patent medicines”[Title/Abstract])).In addition to these sources, grey literature was also within the scope of the search, acknowledging its potential to offer unique and valuable perspectives not found in the indexed literature.

### 2.2 Inclusion criteria

#### 2.2.1 Type of studies

Randomized controlled trials (RCTs), systematic reviews and and meta-analyses involving TCM treatment for DN.

#### 2.2.2 Study participants

Individuals who meet the diagnostic criteria for DN are eligible for inclusion, without any limitations based on age, gender, or disease duration.

#### 2.2.3 Interventions

The treatment group received various forms of TCM interventions, including decoctions, patent Chinese medicines, acupuncture, acupoint sticking, Chinese medicine enemas, integrated TCM nursing care, and exercise therapy, either alone or in combination with standard treatment. The control group was subject to blank control, placebo control, or conventional Western medicine treatments.

### 2.3 Exclusion criteria

Excluded were duplicated publications, articles for which the full text could not be retrieved, and studies with incomplete data, as well as literature reviews, experimental studies, experience reports, and other types of clinical research.

### 2.4 Literature selection and data extraction

Three researchers participated in screening literature and extracting data with cross-verification. Initially, a preliminary screening was conducted on 50 articles, with formal screening proceeding after standardizing the selection criteria. In cases of disagreement, consensus was sought through team discussion or consultation with a third-party researcher.

NoteExpress, a software tool which integrates knowledge collection, management, and application for literature retrieval and management, was used to identify and reject duplications, as well as to consolidate literature information from different databases for summary analysis (31). First, import the retrieved records into NoteExpress and delete duplicates; then, read the titles and abstracts to exclude literature that does not meet the inclusion criteria; finally, read the full texts of the remaining potentially eligible literature to determine the final inclusion, and carry out data extraction. Information from the literature is extracted using a standardized information extraction form, including publication information, study type, grouping method, disease classification, sample size, duration of treatment, Traditional Chinese Medicine (TCM) syndrome patterns, intervention measures, outcome indicators, and efficacy.

### 2.5 Data analysis

Research results are presented through textual descriptions complemented by various forms of graphical data representation, such as tables, line charts, bar graphs, histograms, and bubble charts, to vividly display the distribution and status of key evidence, enhancing readability and facilitating understanding and utilization of evidence (32, 33).

Specifically, line charts represent publication trends; tables display sample size, disease staging, and indicator classification; pie charts exhibit the proportions of TCM syndromes and intervention categories; histograms indicate intervention duration; and heat maps and bubble charts describe evidence distribution, clearly illustrating the quality, sample size, and intervention actions or relationships between multiple variables in included studies on a single three-dimensional graph.

### 2.6 Methodological quality assessment

The included RCTs are assessed for methodological quality using the Cochrane Risk of Bias assessment tool, which encompasses the following main aspects: generation of the random sequence; allocation concealment; blinding of participants and personnel; blinding of outcome assessment; completeness of outcome data; selective reporting of study results; and other potential biases. Each of the studies included is evaluated on these seven criteria to determine whether they pose a high risk, unclear risk, or low risk of bias.The AMSTAR (Assessment of Multiple Systematic Reviews) tool was employed to evaluate methodological quality and assess control for bias in the included studies (34), encompassing 11 items, such as provision of an *a priori* design and comprehensive search strategy. Each item is scored as “yes” (1 point), “no,” “unclear,” or “not applicable” (0 points) according to its criteria, with total scores higher indicating superior methodological quality.

The clinical efficacy in systematic reviews is categorized as follows: “Evidence Uncertain” refers to cases where similar systematic review results are contentious, or the authors consider the conclusions insufficient to constitute definite evidence; “Evidence of No Effect” indicates that the TCM treatment group’s efficacy was equivalent to or worse than the control group; “Evidence of Potential Efficacy” refers to systematic review results demonstrating efficacy but lacking sufficient evidence for a definitive conclusion; and “Evidence of Efficacy” refers to statistically significant efficacy in TCM treatment, with authors expressing no doubt and recommending the method (35).

## 3 Results

### 3.1 Literature search

**Figure 1:**
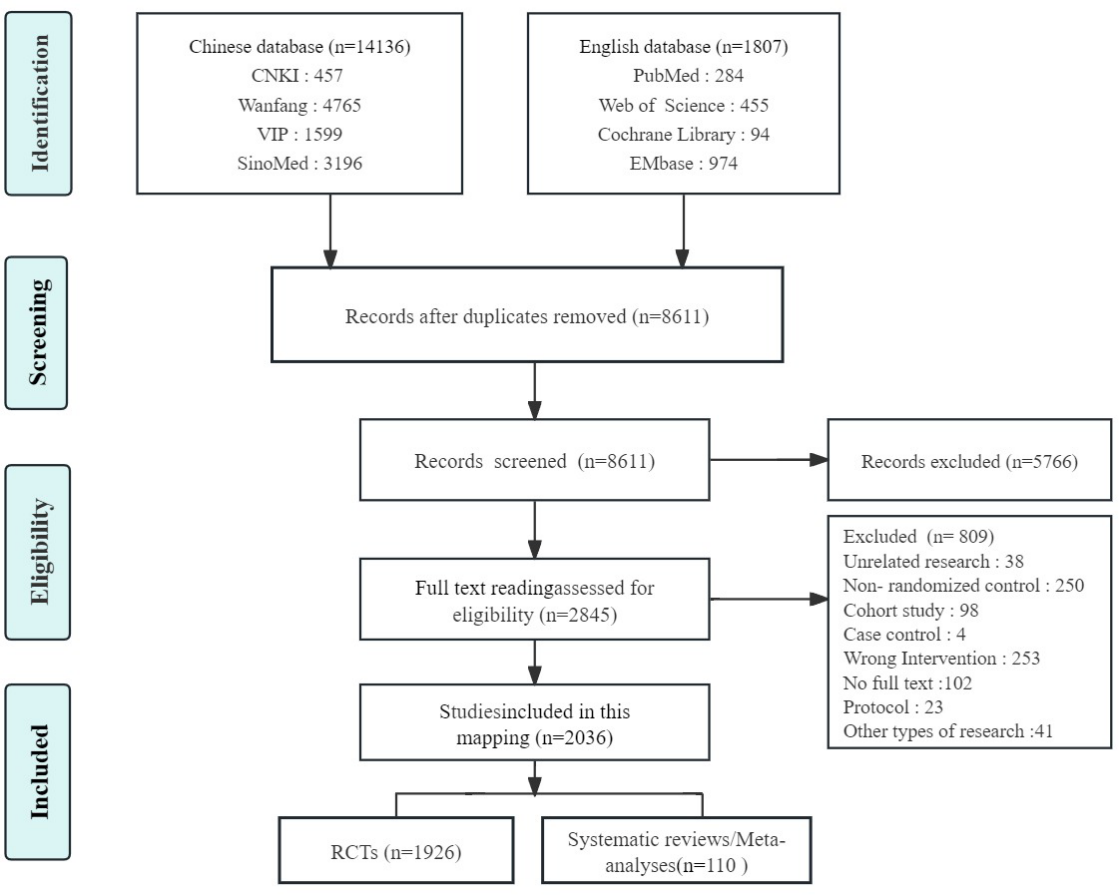
Flow diagram representing the process of literature screening.

A preliminary search of Chinese and English databases yielded 15,943 articles, of which a total of 2,036 were eventually included. This encompassed 1,926 clinical RCTs and 110 systematic reviews/Meta-analyses. The literature screening process is depicted in **Figure 1**.

### 3.2 Publication trends

**Figure 2:**
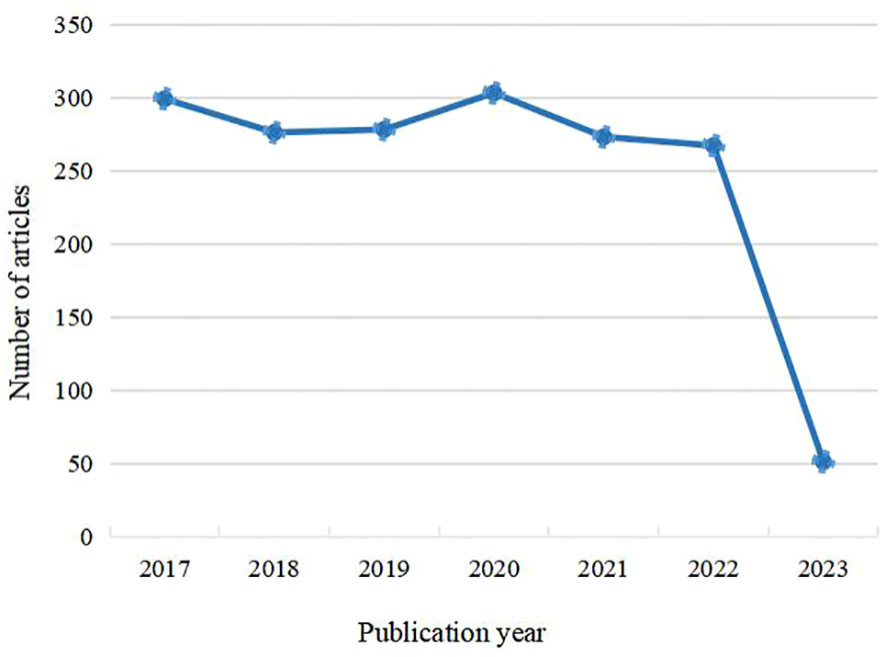
Annual trends in the clinical research literature.

An analysis of 2,036 RCTs and systematic reviews/Meta-analyses on the prevention and treatment of diabetic nephropathy with traditional Chinese medicine, published from January 2016 to May 2023, reveals a generally steady trend in the number of publications. This suggests that traditional Chinese medicine therapies for diabetic nephropathy have consistently attracted widespread attention in recent years, as shown in **Figure 2**.

### 3.3 Characteristics of study population

#### 3.3.1 Staging of diabetic nephropathy

In this study, the staging of included studies was categorized according to the Mogensen staging criteria. When the staging of the study subjects was not explicitly stated in the literature, it was defined as “not stated”. Among them, clinical studies involving patients at stage III of DN were the most numerous, totaling 823 articles, the staging of patients from a total of 466 studies was unclear, as shown in **Table 1**.

**Table 1:** Staging of subjects.

Staging of diabetes nephropathy	Number of research articles (*n*)	Proportion(%)
I	2	0.1
II	1	0.05
III	823	42.73
IV	212	11.01
V	27	1.40
I-II	11	0.57
I-III	40	2.08
I-IV	16	0.83
I-V	4	0.21
II-III	24	1.25
II-IV	11	0.57
II-V	2	0.10
III-IV	264	13.71
III-V	20	1.04
IV-V	3	0.16
Not stated	466	24.19

#### 3.3.2 TCM syndromes

**Figure 3:**
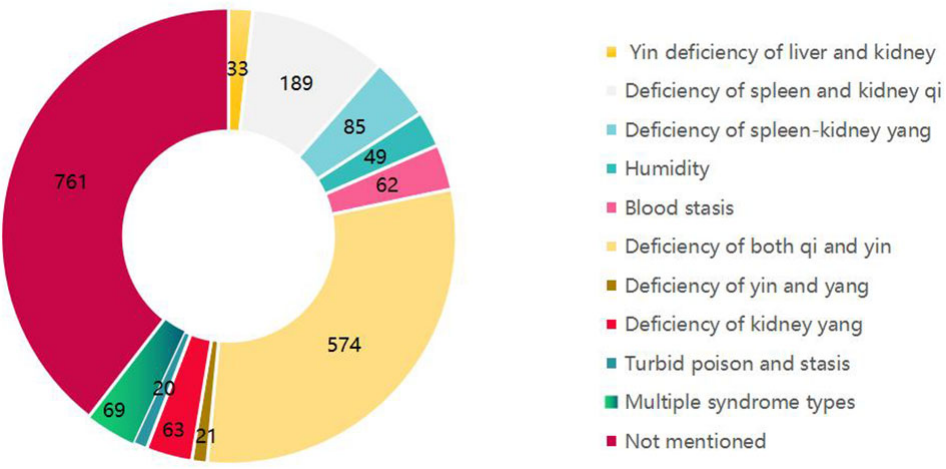
TCM syndrome types in the study subjects.

This study, referencing the “Guidelines for TCM Prevention and Treatment of Diabetic Nephropathy (2011)” (36) and the “Guideline for the Diagnosis and Treatment of Diabetic Nephropathy Integrating Disease with Syndrome” (37), categorized TCM syndromes from the included RCTs. A total of 1,165 studies explicitly specified the TCM syndromes of the study population, with Qi-Yin deficiency syndrome being the most prevalent, as depicted in **Figure 3**.

#### 3.3.3 Sample size

Within the included RCTs, the largest sample size accounted for 1,660 participants, while the smallest encompassed 20. Studies with a participant number ranging from 60 to 100 were the most abundant, constituting 65% of the research, as presented in **Table 2**.

**Table 2:** Sample size.

Study sample size (*n*)	Number of research articles (*n*)	Proportion(%)
*n* < 60	191	9.92
60 ≤ *n* < 100	1246	64.69
100 ≤ *n* <200	444	23.05
200 ≤ *n* < 300	32	1.66
*n* ≥ 300	13	0.67

### 3.4 Treatment regimens and intervention duration

#### 3.4.1 Treatment regimens

TCM intervention measures mainly include nine categories: herbal decoctions, patent medicines, comprehensive TCM care (including diagnostic treatments, diet, emotion management, etc.), acupuncture, acupoint application, herbal enemas, TCM exercise therapy, combination therapies, and other methods (such as acupoint massage, herbal ion introduction, fumigation, foot baths, etc.).

Herbal Decoctions: 1322 articles (68.74%), highest proportion.
Patent Medicines: 356 articles.
Combination Therapies: 125 articles.
TCM Care: 68 articles.
Herbal Enemas: 22 articles.
Others: 11 articles.
Acupuncture: 12 articles.
Acupoint Application: 9 articles.

**Figure 4:**
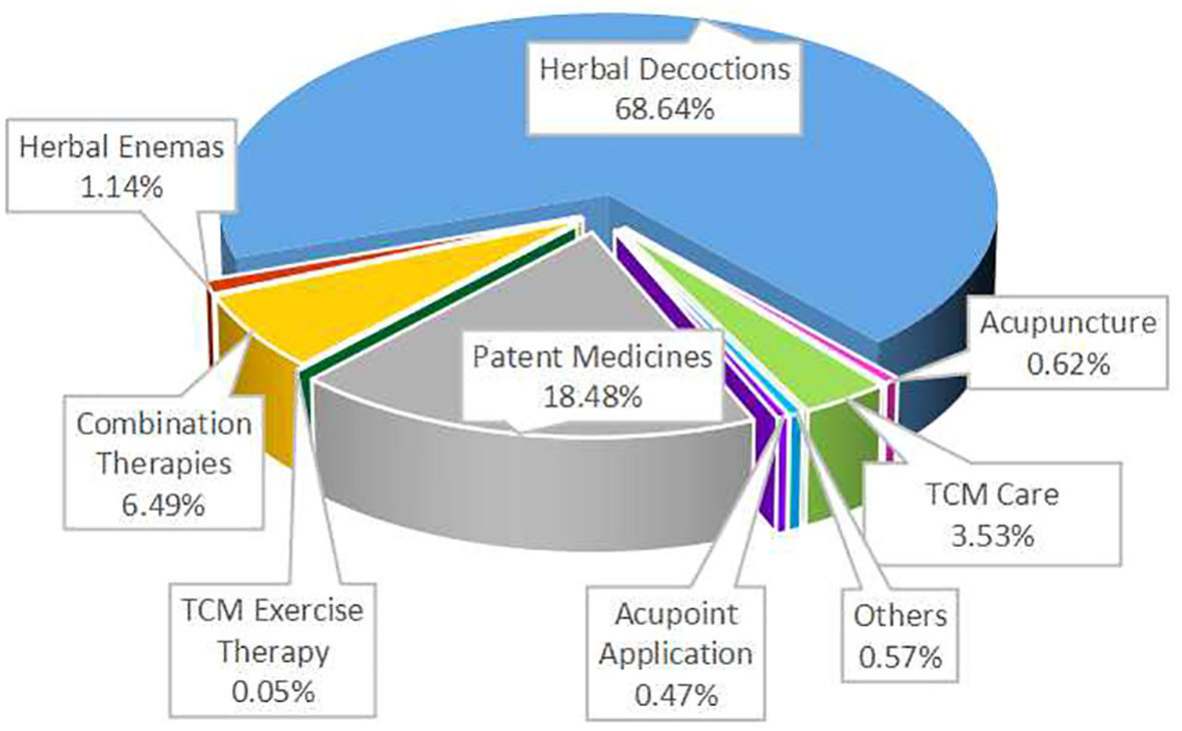
Distribution map of intervention measures categories in RCTs.

TCM Exercise Therapy: Only 1 article, least proportion. Refer to **Figure 4**.

Analyzing the 1926 articles evaluating clinical TCM regimens for DN, 1272 articles (66.04%) utilized TCM diagnostic principles. These either set patients with specific diagnostic patterns as research subjects or provided different interventions or prescription modifications based on varying symptom manifestations. Herbal decoction RCTs mainly used custom prescriptions, focusing on strengthening the spleen and kidneys, nourishing Qi and Yin, and promoting blood circulation. Research on several formulas such as “Yi Qi Yang Yin Huo Xue Fang,” “Yi Shen Huo Xue Tang,” “Yi Qi Huo Xue Tang,” “Tang Shen Fang,” and “Jian Pi Gu Shen Hua Yu Fang” all exceeded 15 items. Studies on decoctions involve 64 classic formulas, with the top 7 most frequently used being “Shen Qi Di Huang Tang,” “Zhen Wu Tang,” “Liu Wei Di Huang Tang,” “Bu Yang Huan Wu Tang,” “Dang Gui Bu Xue Tang,” “Ji Sheng Shen Qi Wan,” and “San Ren Tang.”

Patent medicine studies included oral preparations and herbal injections. Research articles exceeding 5 for specific drugs included 11 oral medications like “Huang Kui Capsules,” “Jin Shui Bao,” “Bai Ling Capsules,” “Dan Hirudo Hypoglycemic Capsules,” and 3 injections: “Huang Qi Injection,” “Shen Kang Injection,” “Dan Shen Injection.” The basic information on the composition and efficacy of herbal decoctions and patent medicines is presented in **Supplementary Tables 1**, **2**.

Combination therapy regimens involved patent medicines combined with decoctions, oral TCM combined with acupuncture or enemas, and comprehensive TCM care combined with other interventions. TCM exercise therapy included “Ba Duan Jin” and “Hu Shen Cao.” Acupuncture treatments embraced conventional needling, moxibustion, and thread embedding, while acupoint application included ear-seed application and herbal acupoint application. Other methods encompassed fumigation, medicinal baths, sealing packages, and soaking, among others.

#### 3.4.2 Intervention duration

**Figure 5:**
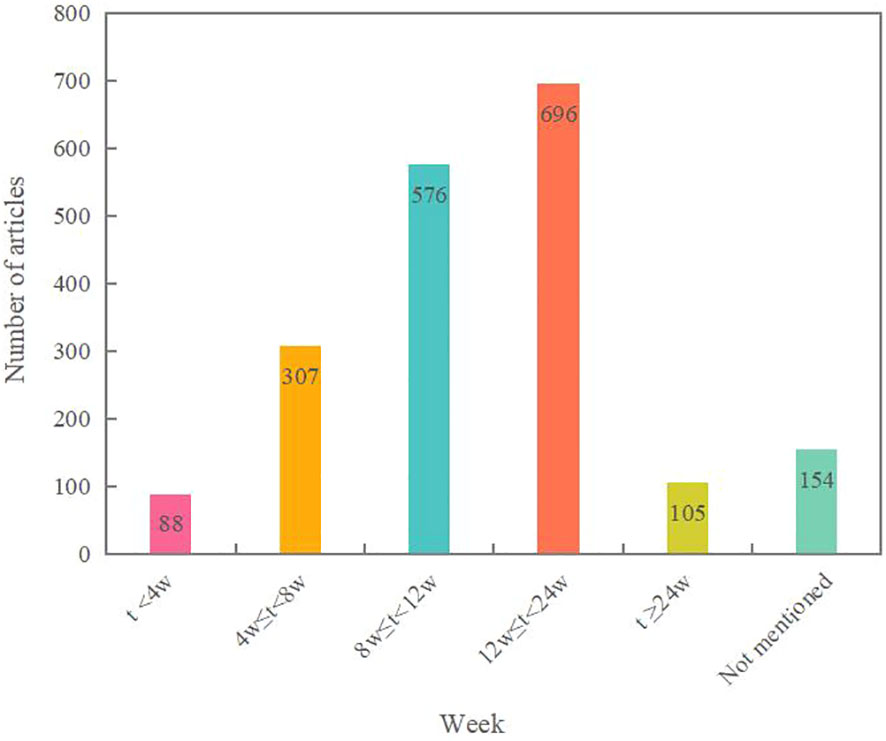
Courses of the TCM control programmes.

The statistical results reveal that the treatment durations are primarily concentrated at 8 weeks and 12 weeks, with 562 and 658 instances respectively. This may be related to the fact that many of the included studies focused on stages III-IV of the disease, and longer intervention durations can be challenging for patients to adhere to. There are 105 studies with an intervention time exceeding six months. See **Figure 5** for details.

### 3.5 Outcome indicators

Research into the therapeutic effects of TCM on DN predominantly utilizes nine categories for evaluating efficacy: ① Clinical effectiveness rate; ② Symptoms and signs, including improvements in clinical symptoms and TCM syndromes; ③ Biophysical examinations, including urine protein, kidney function, renal injury markers, blood pressure, blood sugar, blood lipids, inflammatory factors, coagulation function, hemorheology, endothelial markers, oxidative stress markers, renal fibrosis markers, immune function, and ultrasound imaging; ④ Safety indicators such as routine blood tests, electrocardiograms, and adverse reactions; ⑤ Quality of life and psychological state, using SF-36 life quality score, DQOL (specific life quality scale), SAS (self-rating anxiety scale), and SDS (self-rating depression scale); ⑥ Disease progression and prognosis, focusing on kidney function progression and endpoint event rates; ⑦ Economic indicators, including hospitalization duration and costs; ⑧ Reduction of Western medicine usage; and ⑨ Others, such as treatment satisfaction and compliance.

**Figure 6:**
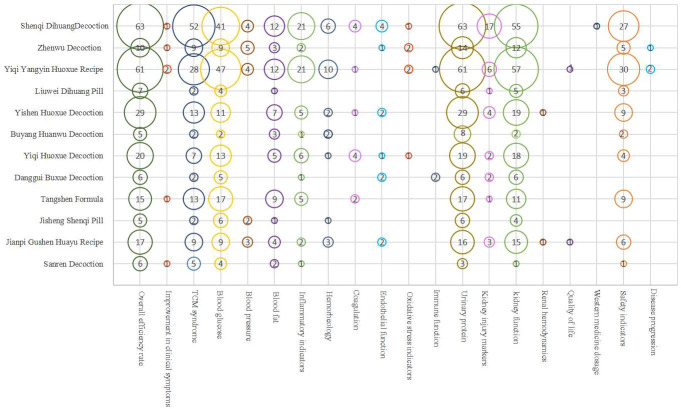
Evidence distribution of outcome indicators of herbal decoctions in treating DN.The x-axis represents the outcome indicator, the y-axis represents the name of the decoction, and the number in the circle represents the number of literature evaluating the outcome indicators of the party.

Bubble chart results reveal that RCTs for DN interventions using herbal decoctions pay significant attention to clinical effectiveness rates, TCM syndromes, blood sugar, urine protein, and kidney function indicators, while less emphasis is placed on renal hemodynamics, quality of life, Western medicine usage, and disease progression (**Figure 6**).

**Figure 7:**
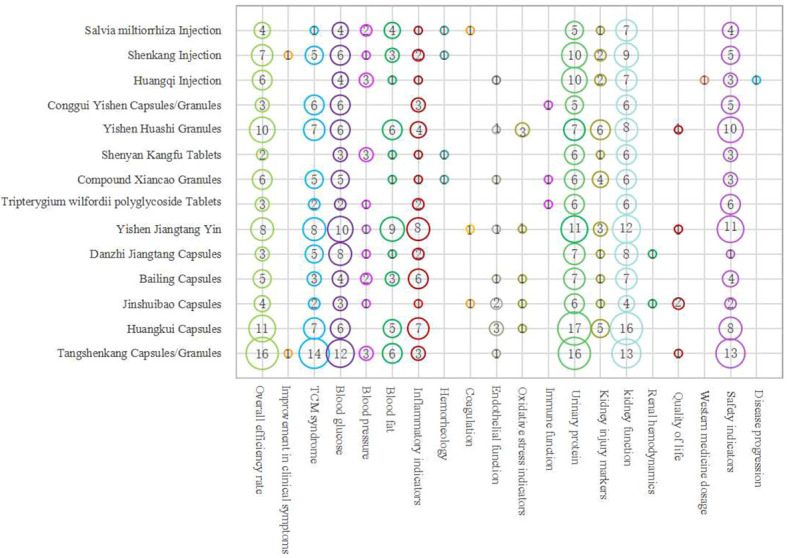
Evidence distribution of outcome indicators of Chinese patent medicines in treating DN.The x-axis represents the outcome indicator, the y-axis represents the name of the drug, and the number in the circle represents the number of literature evaluating the outcome indicators of the party.

Clinical research on patent Chinese medicines for DN treatment generally focuses more on clinical effectiveness rates, TCM syndromes, blood sugar, urine protein, kidney function, and safety indicators, and less on symptom improvement, renal hemodynamics, quality of life, Western medicine usage, and disease progression (**Figure 7**).

Both primarily focus on clinical effectiveness rates and biophysical indicators, wherein clinical effectiveness rates amalgamate evaluations of biophysical objective indicators with subjective indicators reflecting symptom improvement, effectively providing a comprehensive clinical efficacy evaluation.

Although TCM treatment for DN has significant advantages in improving renal function and clinical symptoms, differences in outcomes between studies exist, potentially related to treatment duration, medication dosage, dosage form, and individual differences. The dosage of the prescription directly affects the clinical efficacy of TCM, with the same medicinal composition having vastly different effects due to dosage variations; even a single herb in a formula can have significantly different effects depending on its dosage (38). The clinical efficacy of medicines also varies depending on the method of preparation. Differences in treatment duration across studies can introduce measurement bias. The selection of outcome indicators is influenced by subjective factors of both researchers and subjects, posing a risk of not fully objectively reflecting clinical efficacy; furthermore, the outcome indicators selected in the included literature are not entirely the same, leading to publication bias, potential reporting, and missing data issues (39). These factors may all contribute to heterogeneity in results, affecting the scientific validity of the research. Future studies should consider multiple influencing factors and include more literature for subgroup analysis among multiple groups to reduce heterogeneity between studies, aiming for a more objective evaluation of the clinical efficacy of TCM treatment for DN.

### 3.6 Methodological quality assessment

**Figure 8:**
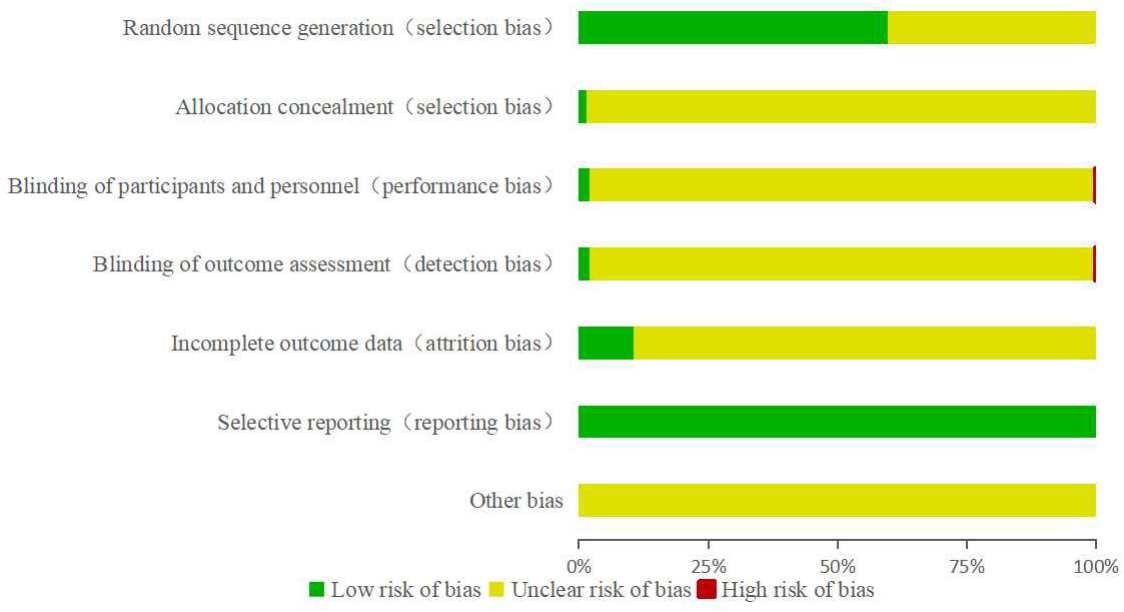
Bias analysis of RCTs on traditional Chinese medicine in treatment of DN.

Among the 1,926 RCTs included, approximately 59% employed reasonably sound random allocation methods, classified as low bias risk. These methods included the use of random number tables, Excel rand functions, and SPSS random grouping. The remaining studies merely mentioned randomization without detailing the specific plan, thus the risk of bias was unclear. Only 27 articles used envelope concealing schemes, considered to be at low risk of bias; the rest did not describe any concealing scheme. Thirty-eight articles detailed specific blinding procedures, two studies were non-blinded, evaluated as high risk of bias, while the others did not specify any blinding methods. Regarding data integrity, about 10% of the studies reported cases of loss to follow-up, dropout, and other similar issues, indicating low risk of bias. All studies reported outcome assessment indicators listed in the study methods, without clear mention of other potential biases (**Figure 8**). The overall quality of the included literature was not high, and while the integrity of result data was relatively satisfactory, most studies did not mention allocation concealment, implementation of blinding, evaluation of blinding, or other bias situations, indicating a large number of studies with unknown bias risks. Therefore, further standardization of RCT designs for TCM treatment of DN is required to provide high-quality trial data for clinical use.

**Figure 9:**
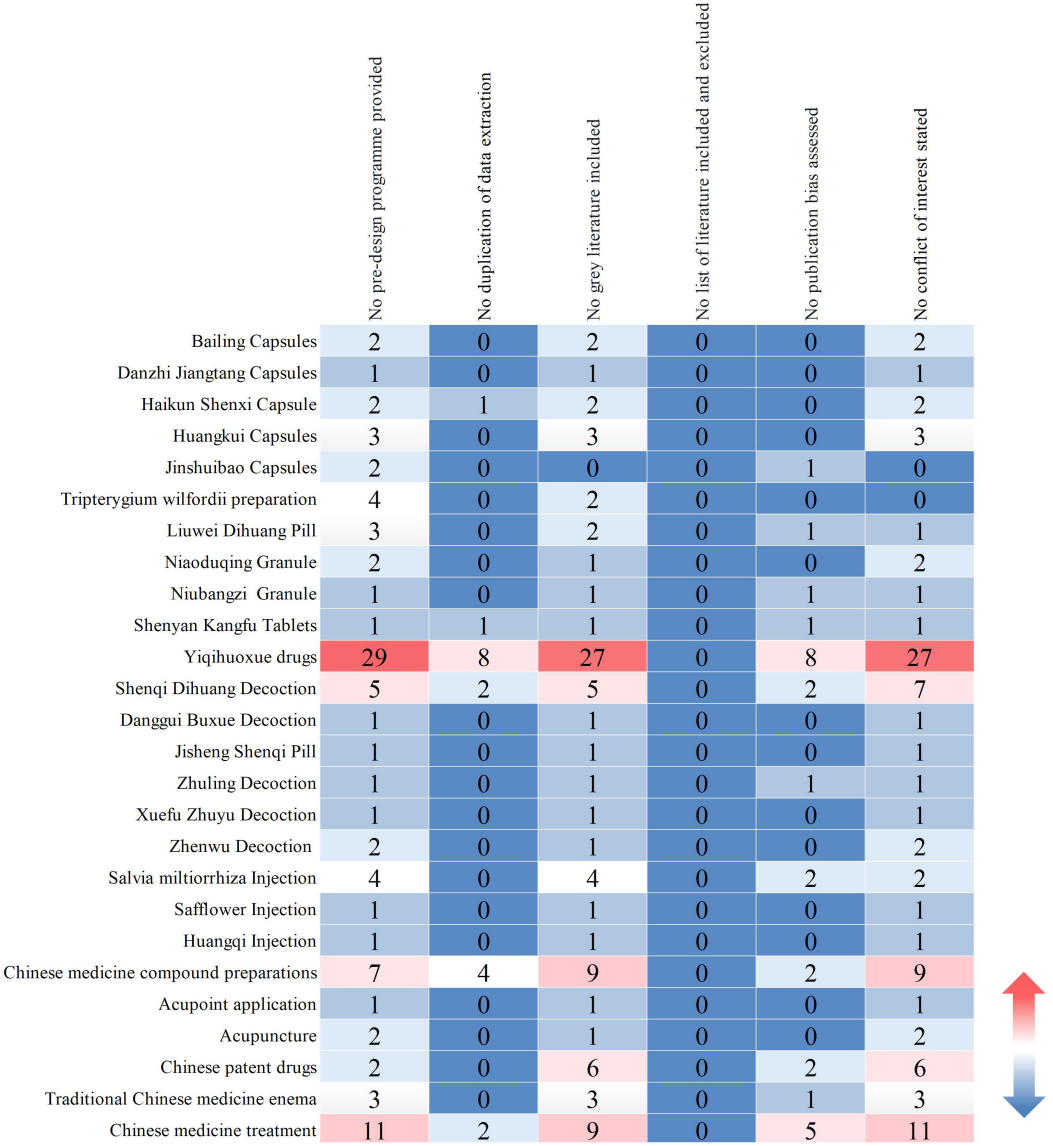
Distribution of evaluation items for Systematic reviews/Meta analyses.

In this study, a total of 110 Systematic reviews/Meta analyses were included, based on the types of TCM interventions. These encompassed: 49 articles on herbal decoctions; 32 articles on oral and injectable patent medicines; 9 articles on compound preparations of Chinese medicines; 6 articles on non-pharmacologic therapies (enemas, acupuncture, acupoint patching); and 14 articles on comprehensive TCM therapies. Evaluating the methodological quality of the included studies revealed that none of them provided a complete list of included and excluded literature. There were 93 articles that did not provide preliminary design schemes; 18 articles exhibited a lack of redundancy in data extraction; 87 articles did not consider the retrieval and inclusion of grey literature; 27 study reports did not evaluate the possibility of publication bias; and 89 study reports did not provide declarations of conflict of interest (**Figure 9**).

**Figure 10:**
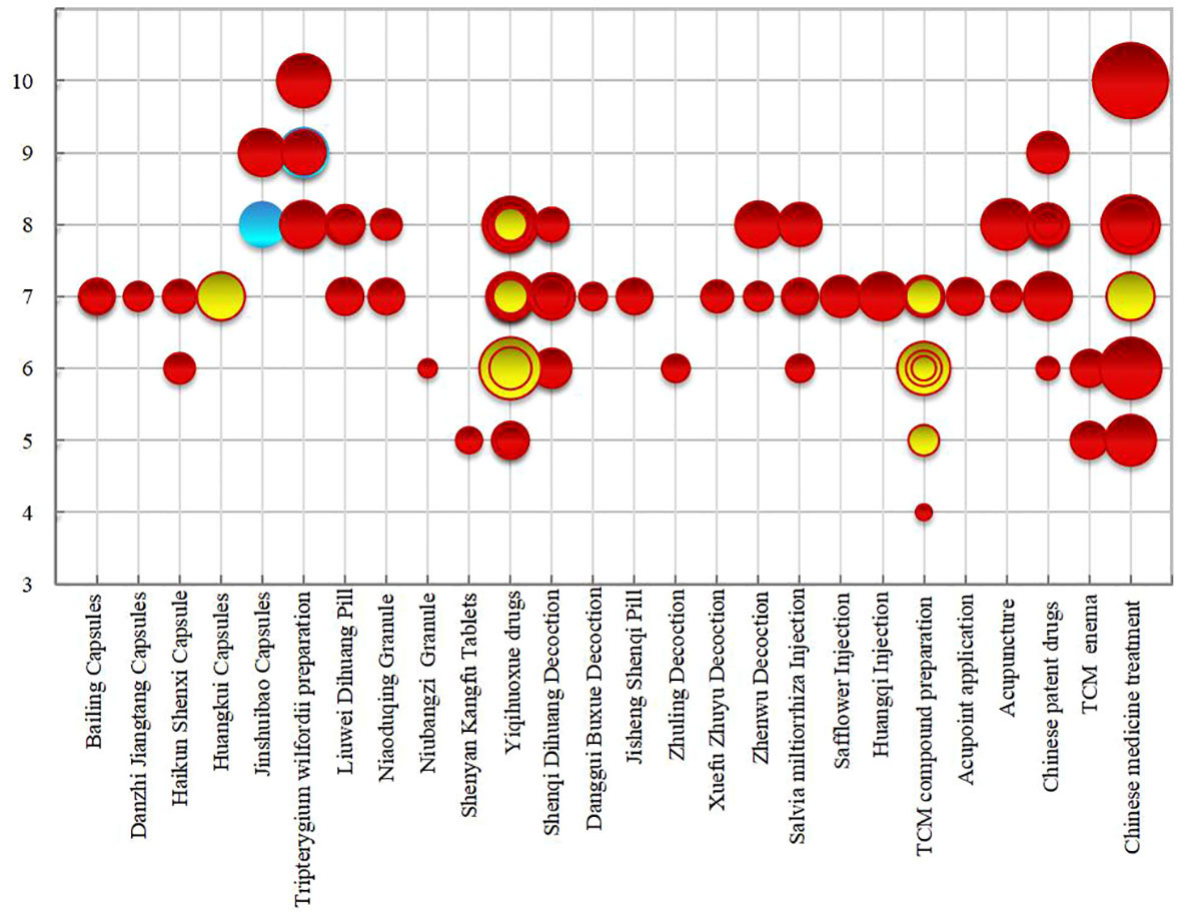
Methodological quality evaluation of Systematic reviews/Meta analyses.

The bubble chart (**Figure 10**) results show that among the included literature quality scores, 2 articles scored 10 and 4 articles scored 9, classified as high-quality literature; 1 article scored 4, considered low-quality; articles scoring between 5 and 8 were of medium quality, with 70% of the studies scoring between 7 and 8. The overall methodological quality assessment of the included studies was relatively low, primarily due to: the lack of pre-study protocol development and registration, which could lead to reporting bias; failure to provide excluded literature, reducing the rigor of the research; incomplete literature searches, not clarifying research-related conflicts of interest, decreasing the credibility of the research, and leading to potential publication bias. The majority of conclusions suggested potential efficacy, with 14 articles showing effectiveness and 2 articles showing unclear efficacy, with no conclusions indicating ineffectiveness. In summary, most systematic reviews/meta-analyses included original research of limited quality and quantity, with methodological flaws, failing to reach definitive conclusions on long-term efficacy. Future research should focus on the registration of study protocols, supplement searches in professional databases and grey literature, standardize research processes, and improve research quality to increase the credibility of the study conclusions, providing stronger support for evidence-based conclusions.

The x-axis represents the evaluation items, and the y- axis represents the intervention measures of TCM. The change in color of “blue-white-red” represents the number of research literature from low to high, and the number represents the corresponding number of literature.

The x-axis represents the intervention measures, the y-axis represents the methodological quality score, the size of the circle represents the number of included literature, and different colors represent different efficacy conclusions.Red indicates evidence of potential evidence of Systematic reviews/Meta analyses, yellow indicates effective evidence of Systematic reviews/Meta analyses, blue indicates unclear evidence of Systematic reviews/Meta analyses.

## 4 Discussion

This investigation provides a comprehensive review of clinical RCTs and systematic reviews/meta-analyses of TCM in treating DN since 2016. Through evidence mapping, data on publication trends, preventive measures, and outcome measures, among other factors, were analyzed and presented. The intent is to encapsulate the current state of TCM research in treating DN, offering insights for the future design and optimization of clinical studies in TCM. The primary observations based on the current evidence are as follows:

### 4.1 Quality of clinical RCTs needs enhancement

While a considerable number of RCTs have been executed, nearly 65% of these studies possess a small sample size, mainly ranging between 60 to 100 participants. The typical treatment duration lies between 12 to 24 weeks, indicating a relatively brief intervention period. As a consequence, the long-term efficacy of TCM might not be sufficiently demonstrated or even discerned (40). Continuous outcome assessment studies across various stages of DN are of pressing importance. All examined studies referenced random grouping, yet half of the RCTs did not clarify the specific randomization methods. Of the 1,150 studies that described randomization methods, a significant majority didn’t report the rationale for their sample size determination. They also neglected to describe aspects like allocation concealment, blinding settings, or drop-outs. Such design deficiencies might introduce bias, possibly affecting the reliability of the study findings (41). Thus, future research endeavors should lean towards more extensive, double-blind RCTs with prolonged follow-up durations. Adhering to proper trial design and aligning with the CONSORT statement (42) will facilitate the provision of substantial evidence-based information for clinical practice.

### 4.2 Precision in disease staging is imperative

Approximately 76% of the included studies specified staging criteria, encompassing the Mogensen stages and the GA staging standards released by the Kidney Disease: Improving Global Outcomes (KDIGO) in 2012 (43). However, there is a noticeable absence of a unified staging standard.DN’s prevention and treatment follow a continuous trajectory. Accurate staging enables crafting interventions aligned with the condition’s severity. Ambiguities in disease staging can potentially usher in overtreatment or undertreatment scenarios, compromising drug efficacy. Such inconsistencies present hurdles for future guideline formations, consensus development, and their subsequent influence on clinical practices.

Nearly 20% of the RCTs confined their subjects to patients across multiple disease phases but refrained from basing their efficacy analyses on these stages. Such an approach might cause the study results not to mirror the disease’s severity or the therapeutic efficacy accurately (44). Studies that amalgamate patients across different stages can be counterproductive when merging data for meta-analyses, creating avenues for heterogeneity (45, 46).

Given the swift progression of DN, varying diagnostic markers emerge at different disease stages. Distinct differences in the degree of target organ damages, symptom complexities, and therapeutic objectives are observed. As such, consistent staging principles and therapeutic strategies become vital. These can effectively guide and standardize clinical practices, aiming to hone the therapeutic efficacy of TCM in addressing DN (47).

### 4.3 The Diagnostic and therapeutic philosophy of TCM must be fully emphasized

The principle of “Bian Zheng Lun Zhi”–which involves diagnosis and treatment based on an individual’s specific symptoms and signs – embodies the essence of TCM. This approach integrates and analyses the symptoms and signs gathered through the four diagnostic methods: inspection, listening and smelling, inquiry, and palpation. A treatment strategy is then determined based on the identified syndrome pattern (48, 49). In this context, depending on the patient’s constitution and disease stage, different treatments might be prescribed for the same disease with different syndrome types, or for different diseases with the same syndrome type, showcasing the uniqueness and strength of individualized treatment in TCM (50, 51). The syndrome, pivotal to this process, links the diagnosis and treatment and serves as a key aspect in integrating disease patterns with symptomatology (52).

However, in the included RCTs, 40% did not specify the TCM syndrome type for the study participants, and about 53% did not use TCM disease and syndrome indicators as outcomes to evaluate clinical efficacy. Currently, multiple classifications exist for diagnosing diabetic nephropathy in TCM, and there’s a lack of uniform diagnostic criteria and objective evaluation indicators, limiting the comparability and reproducibility of TCM syndrome-specific treatments. Future research should prioritize the identification and assessment of TCM syndromes, while also refining integrated treatment strategies that combine both Western and traditional Chinese medicine.

Research (53) suggests that an integrated model, which combines macro-level syndrome differentiation with micro-level indicators, offers a precise and effective method for TCM treatment of diabetic nephropathy. This model enables accurate diagnosis of the disease and syndrome-targeted treatment. Therefore, subsequent studies should leverage systems biology to explore the common material basis at structural or functional levels in both TCM and Western medicine (54). This can help in optimizing the syndrome scale, elucidating the essence of syndromes, and laying the foundation for an integrated diagnostic and therapeutic approach to diabetic nephropathy.

### 4.4 Challenges and advancements in the early diagnosis of DN and evaluation of TCM symptomatology

Effective early intervention strategies are pivotal to mitigating the risk of adverse outcomes and the associated disease costs, with timely diagnosis being paramount. Extensive research and clinical evidence underscore the challenges in early diagnosis of DN. Commonly utilized markers, such as the urine albumin-to-creatinine ratio, can be greatly influenced by factors like exercise and fever, and exhibit considerable inter-individual variability (55). These markers often lack specificity and sensitivity (56, 57). Moreover, DN’s onset can be insidious; by the time these markers decline, optimal intervention opportunities may have been missed (58). Notably, tubular damage is present in the early stages of DN and closely aligns with disease progression (59, 60). This damage serves as an indicator for early detection and monitoring of DN progression. Recent years have witnessed a surge in studies on biomarkers for early DN, but many such studies have small sample sizes, are cross-sectional in nature, or show limited results due to decomposition factors affecting the detection of urinary neutrophil gelatinase-associated lipocalin (NGAL) and kidney injury molecule-1 (KIM-1) at times of tubular injury (61). Therefore, larger prospective clinical trials are essential to further evaluate their predictive value, aiming to identify sensitive and reliable predictors, providing a window for early intervention and averting or delaying the onset of irreversible complications, thereby minimizing severe cardiac and renal morbidity and mortality (62).

Furthermore, although efficacy rates and TCM symptom scores originate from roughly the same sources, the specific standards set differ, leading to underutilization of the data. TCM symptomatology, unique to Chinese medicine, is based on a four-diagnosis symptom scale, reflecting changes in symptomology and signs constituting the syndrome before and after treatment. Despite widespread use, several shortcomings exist in the evaluation methods for TCM efficacy. There lacks a unified standard for its connotation and assessment, often focusing solely on primary symptoms of a syndrome type without distinguishing between subjective and objective content. This can undermine the content validity of evaluations. Assessments are also prone to observer bias, compromising test-retest reliability.

### 4.5 Outcome indicators should be more representative

In current research, the utilization of efficacy rates, renal function, and blood glucose markers exceeds 50%. However, key outcome indicators, such as renal injury biomarkers, quality of life, economic evaluations, and clinically relevant outcomes, have been under-emphasized.

DN is a progressive renal disease characterized by recurrent symptoms and prolonged treatment cycles. Economic considerations are increasingly recognized in evaluating its therapeutic outcomes. As the disease progresses, patients experience a decline in quality of life (63) and see a significant surge in healthcare costs and disease burden (64). Inadequate treatment can lead to renal insufficiency, advancing to end-stage renal disease, which in turn escalates the risks of cardiovascular complications, renal failure, and mortality (65). Hemodialysis, the primary treatment in this stage, effectively prolongs patient survival but demands high patient compliance and incurs substantial costs. Additionally, patients frequently experience psychological issues like depression and anxiety, severely affecting prognosis and quality of life (66). Health economic evaluations are a crucial aspect of national health insurance reform policies (67). Incorporating health economic indicators in clinical research assists clinicians in making informed clinical decisions and aids patients in selecting the most appropriate treatment modalities (68).

In treating DN, TCM primarily emphasizes delaying renal failure and gives priority to clinical outcome assessments over merely relying on biochemical markers. Long-term therapeutic outcomes are its crucial evaluation metrics. Most RCTs focus on Stage III patients, who typically show minimal symptoms, incur lower treatment costs, maintain stable quality of life, and participate in short-term clinical trials. Rarely do these studies focus on long-term clinical endpoint events, necessitating extended follow-ups to gauge patients’ life quality. Such outcome deficiencies can impact the evaluation of the long-term effects of herbal medicines and the reliability of research findings, hindering a comprehensive assessment of TCM’s advantages in treating DN. To better evaluate the efficacy and reliability of TCM in treating DN, future research should establish a core outcome set for DN (69), select more representative evaluation indicators, and place greater emphasis on patient quality of life, economic conditions, and endpoint outcomes. This approach will allow a more holistic assessment of drug clinical efficacy and safety.

## 5 Limitations and future perspectives

For the first time, this study employs an evidence map to elucidate and showcase the current state of systematic reviews/meta-analyses and clinical research on TCM treatments for DN. The findings indicate that TCM holds certain advantages in the treatment of diabetic nephropathy. However, there remains room for improvement in our research.

This search was limited to Chinese and English databases post-2016, leading to a single study type and somewhat restricted evidence. Future endeavors could encompass a broader range of databases, clinical trial registries, and web sources, with periodic updates to research data. The overwhelming majority of studies included were conducted in China, thus limiting the generalizability of the results. The quality of evidence from original studies was not thoroughly evaluated, posing a risk of an incomplete evidence distribution assessment. During literature screening, we noted instances of split publishing of research findings, which could potentially influence aggregate results. There was variability in the types of medications, dosages, and treatment durations for each intervention, which introduces some limitations to the applicability of the study outcomes.

In recent years, there has been a rising incidence of DN, and consequently, the rate of ESRD has been increasing (70). It is projected that by 2030, the global demand for hemodialysis and kidney transplantation will double, placing substantial strain on healthcare systems (71). Therefore, effective prevention of DN onset, slowing the progression of renal function decline, reducing the incidence of endpoint events, improving the quality of life for dialysis patients, decreasing cardiovascular event rates, and harnessing the significant therapeutic advantages of TCM in DN prevention, progression, and comprehensive treatment are pressing issues that need to be addressed in the clinical management of DN.

## 6 Conclusion

Future research should focus on addressing the limitations identified, with a particular emphasis on the clinical challenges associated with DN. There is a critical need for conducting high-quality clinical trials that enhance the credibility of evidence for practical application. Establishing clinically relevant, standardized outcome measures is essential, incorporating multiple indicators for a comprehensive assessment of efficacy. Integrating the TCM concepts of disease and symptom patterns can significantly improve the practicality and research potential of therapeutic evaluation metrics.

A crucial element in clinical research involves leveraging emerging evidence-based medicine and clinical research techniques for indicator selection. This process should account for the disease characteristics, study objectives, and intervention properties while fostering innovation, practicality, sensitivity, and specificity. The adoption of sensitive measurement time points aims to identify indicator patterns and systems that resonate with TCM’s core principles. The development of a Core Outcome Set for TCM Treatment of DN (CMOS) intends to offer a standardized set of essential indicators for trial reporting. This initiative will not only facilitate study comparisons and result integration but also mitigate the heterogeneity in outcome indicators across similar studies, thereby enhancing the utility of research evaluation metrics and the overall value of research outcomes.

Incorporating advanced techniques such as genomics and bioinformatics into TCM research represents a significant step forward. This approach not only modernizes TCM research but also provides insights into the active components, their target actions, and the underlying molecular mechanisms of TCM treatments. Integrating these precision tools can refine the accuracy and effectiveness of TCM interventions. Embracing real-world evidence and adopting proven evaluation methodologies will strengthen the evidence base, enriching the TCM therapeutic arsenal for managing diabetic renal diseases.

Ultimately, the goal is to elevate the scientific validity and efficacy profile of TCM through evidence-based clinical research. By embracing these strategies, future research can overcome existing hurdles and unlock new possibilities for the effective treatment of DN, ensuring that TCM’s rich heritage is harnessed to meet contemporary healthcare needs.

## Data availability statement

The raw data supporting the conclusions of this article will be made available by the authors, without undue reservation.

## Author contributions

YG: Writing – original draft, Writing – review & editing. ZL: Writing – original draft, Writing – review & editing. YW: Writing – original draft, Writing – review & editing. HZ: Writing – original draft, Writing – review & editing. KH: Writing – review & editing. YF: Writing – original draft. SX: Writing – original draft. QL: Writing – original draft. XL: Writing – original draft, Writing – review & editing. GZ: Writing – review & editing.
